# The five deadly sins of science publishing

**DOI:** 10.12688/f1000research.6488.1

**Published:** 2015-05-11

**Authors:** Vitek Tracz

**Affiliations:** 1 Faculty of 1000, London, W1T 4LB, UK

**Keywords:** Open Access, Open data, Open Peer Review, F1000, Life Sciences, Publishing

## Abstract

Science cannot progress without scientists reporting their findings. And yet researchers have given control of this central pillar of the scientific process to science publishers, who are in the business of serving the interests of their journals; these are not always the same as the interests of science. This editorial describes the problems with the process of preparing and publishing research findings, and with judging their veracity and significance, and then explains how we at Faculty of 1000 are starting to tackle the ‘deadly sins’ of science publishing.

## I was a sinner

I was, for many years, a typical science publisher, taking advantage of an unusual set of circumstances and making money while not helping (possibly even hurting) science, even though at the time I did not quite grasp how much. Then, when the web arrived in the 90s, I had a partial conversion. The web made many things possible and we started by creating an online community for scientists (BioMedNet) and an associated web magazine (HMS Beagle).

Soon afterwards, we realised that it was now possible and important to open up access to research articles that were normally locked away behind subscription barriers. There was a small group of key individuals involved in launching the first open access publisher, BioMed Central and the first open access repository, PubMed Central in the year 2000. I was the only publisher in that group; the others were scientists, including David Lipman (of NCBI and the inventor of PubMed), Harold Varmus (the then head of NIH), and Pat Brown and Mike Eisen (both outstanding scientists). We all took a very big risk and were attacked by many scientists, scientific societies, journals, publishers and others for the open access approach. It still surprises me that the whole scientific community accepted and supported (or simply paid no attention to) a longstanding state of affairs that has made accessing the findings from other’s research difficult or impossible (and expensive) for many scientists, as well as for others who are interested in them.

I started to realise how central a role science publishers play in the life and inner workings of the scientific community. It is a truism to say that science cannot exist without scientists reporting their findings, and these findings being used to develop other’s research; as it is often said, we stand ‘on the shoulders of giants’. Through the combined efforts of hundreds of thousands of researchers finding truths – large and small – science moves forward towards an ever greater understanding of the world in which we live. Yet researchers have given control of this central pillar of the scientific community to science publishers, the journals they publish, and the professional editors they employ, some of whom are in the business to serve the interests of their journals; these are not always the same as the interests of science.

I am pleased to say that the concept of open access is finally gaining ground, and I do believe it will, in time, become the norm in life science publishing. This is due (in no small part) to the funders who decided to support it, and in this there may be a lesson for the future. However, the success of open access only addresses one of many ‘sins’ that plague the science publishing industry. In this editorial, I will describe the problems involved in the process of preparing and publishing research findings, and in judging their veracity and significance, and try to explain the ways in which my colleagues and I in the Faculty of 1000, alongside over 11,000 scientists (as F1000 Faculty Members), are starting to tackle the other ‘deadly sins’ of science publishing.

## The five deadly sins

### Sin 1: Delay

**Figure 1.**
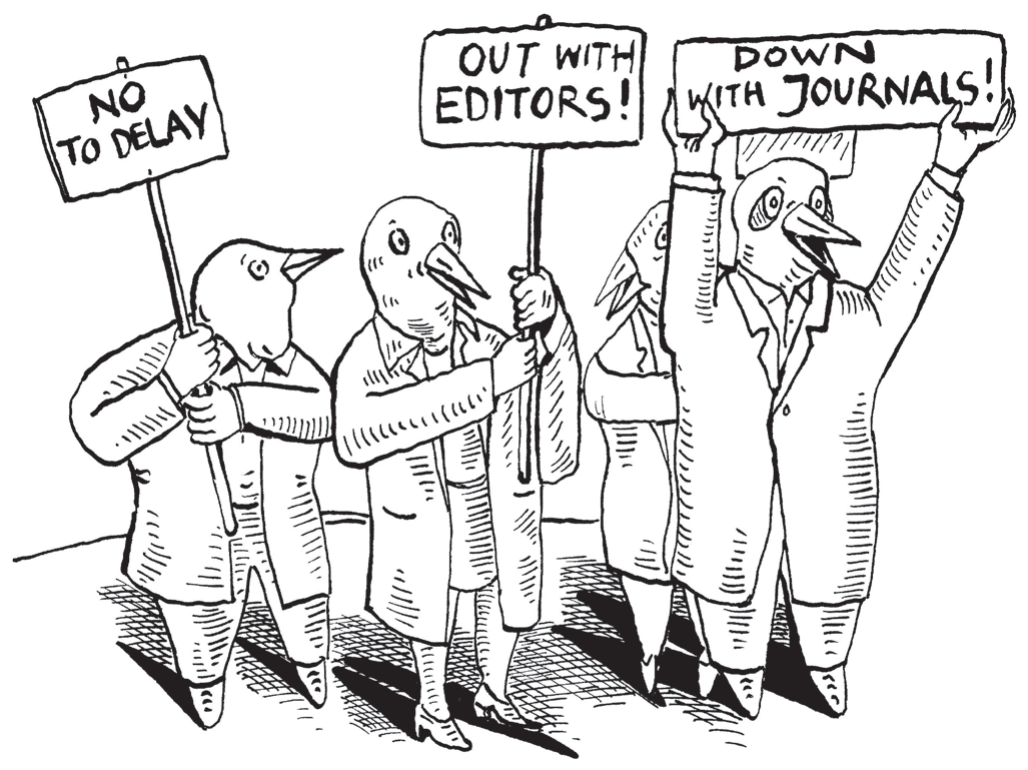
Cartoon of demonstrations that would seem appropriate outside journal offices.

I find it incredible that researchers who wish to publish findings in the fields of biology and medicine are accustomed to a delay that regularly runs anywhere from 6 months to 1 year before their results become public. Who benefits from this delay? Why is no one complaining? Findings that might be useful in research today will be all too often hidden for an inexcusably long time. Why are there no demonstrations of scientists in front of journal offices with placards saying “No to Delay”, “Down with Journals”, “Out with Editors” ( Figure 1)? Because it is the existence of the journals themselves, the role of academic editors, and the processes that they operate in secret that are responsible for this delay. Of course, even open access journals can be just as guilty of this as traditional, closed access journals.

### Sin 2: Journals and their editors

Scientists should not need journals or editors to decide what should be published. Scientists read articles, not journals. Current search methods, encompassing Google Scholar, PubMed and many others, generally enable us to find the article that we want to read very rapidly, and search technology continues to improve as more industries switch to the digital medium. PubMed looked some time ago to see whether there was a correlation between the decision of a searcher to open an abstract (thus indicating an interest in the article) and the Impact Factor of the journal. They were surprised to find that the probability of clicking on an article from a journal was strongly correlated with the number of articles published by the journal per unit of time, rather than with the journal’s Impact Factor. Of course this makes sense – the reader scans the list of citations in a search result looking for titles that suggest they might contain something relevant to their question, and often does not even consider in which journal they are published.

Following the advent of the web, journals ceased to be important for readers but maintained importance for authors who wanted to get the reflected benefit of the Impact Factor (I will come to this poisonous issue later), which can play a major role in deciding their future as a scientific researcher. As a result, researchers and authors voluntarily relinquish control over this process (and consequently also over the public discourse on science as a whole) to journals and their editors who make decisions for their own reasons.

The editors who exert control over the publication process decide what and when to publish (or perhaps more often, what not to publish), but may not always be experts in the specific topic of the individual paper to really make this decision. They rely on the undisclosed advice of secretly appointed referees, who may do their work poorly (nobody but the editor will know), and may have obvious conflicts of interest (for example, they might be a direct competitor).

### Sin 3: Peer Review is broken

It has become an oft-mentioned complaint that the peer review system is ‘broken’. It surprises me, then, that no one seems to be in a rush to decide exactly what component is broken and how to fix it. Perhaps the main problem is recognising who peer review is for. Currently, in most cases, peer review is done for journal editors to help them decide what not to publish. Authors need and benefit from peer-review, which at its best is one of the most amazing examples of a community-wide, altruistic action. And yet in most cases, the authors do not know who is reviewing their work; they learn of the comments through the editors without necessarily knowing the context of the comments; and will often be refused publication even when reviewers have useful comments and consider the work good.

**Figure 2.**
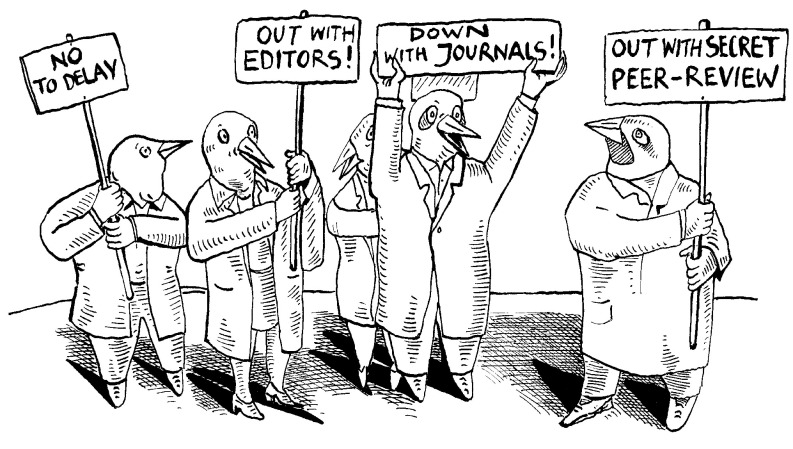
Cartoon of further demonstrations that would seem appropriate outside journal offices.

In addition, the reviewers’ comments often provide a useful commentary on the work and related research, are well written and can sometimes contain useful insights, that under the traditional peer review system may be partially censored before being seen by the authors and which are rarely available to the reader. The often significant efforts of reviewers disappear, and the reviewer gets little direct benefit for their work. Why do researchers accept it? Let’s add a placard to the demonstration in front of the journal offices: “Out with secret peer review” ( Figure 2).

### Sin 4: Where are the data?

We are facing a potential crisis of irreproducible results. Many research articles do not include sufficiently detailed materials and methods, and lack the data necessary to be able to judge the analysis that led to the conclusions that have been made. Including the data on which results are based within the narrative of an article is difficult for authors and for publishers. Most publishers do not insist, or are prepared to publish partial data, often in a difficult-to-use form. Often referees make recommendations about an article without having practical access to the relevant data. The reproducibility problem is complex and it will be a major challenge. Providing complete data and details of the way it was obtained has to be a part of this task.

### Sin 5: The Impact Factor

Behind the inappropriate and unfair way the peer-review process is conducted by journal editors lies a reason – the journals fight as hard as they can to increase their Impact Factor. This metric was invented by a great innovator in library science, Eugene Garfield, who founded Current Contents, Citation Index and the infamous Impact Factor. He meant it to be a tool for science librarians as a measure of the journal’s total citations, and was not meant to say anything about the quality of an individual article. He now openly hates its misuse.

The Impact Factor has become an easy way for committees deciding on a scientists’ future to judge the standard of their research. Everyone involved in the research ecosystem has now become slaves to it, even though many feel it is unfair, misleading, imprecise, and just plain wrong. The recent San Francisco Declaration on Research Assessment ( DORA, 2013) echoed these sentiments and was signed by many reputable scientists, journals and institutions, but nothing has changed. The fact that using the Impact Factor is the wrong way to decide people’s research funding, jobs and careers is now almost universally accepted. Yet despite that, it is still in use, whether used in the capacity of a scientist being judged by this approach, or by someone sitting on the judging committee. How can we explain this?

To impede the universal use of the Impact Factor will be very hard, and will require strong decisions from the bodies governing and funding science. It will also require the development of alternative habits and tools to make this assessment fair and not overly onerous. One thing is certain; it must directly involve the assessment of the specific scientific achievements of individual researchers by people with appropriate expertise.

## Overcoming the sins

There are of course many who have attempted – and who continue to try – to address one or other of these sins in an admirable way. However, as far as I know, no one until now has taken up the task as a whole, and many groups working on parts of the task are small and poorly funded. We have whole heartedly taken on creating an industrial-scale, integrated solution and we have the stamina for a long battle. We have survived the struggle to promote and disseminate open access, and we will survive the other struggles to come.

**Figure 3.**
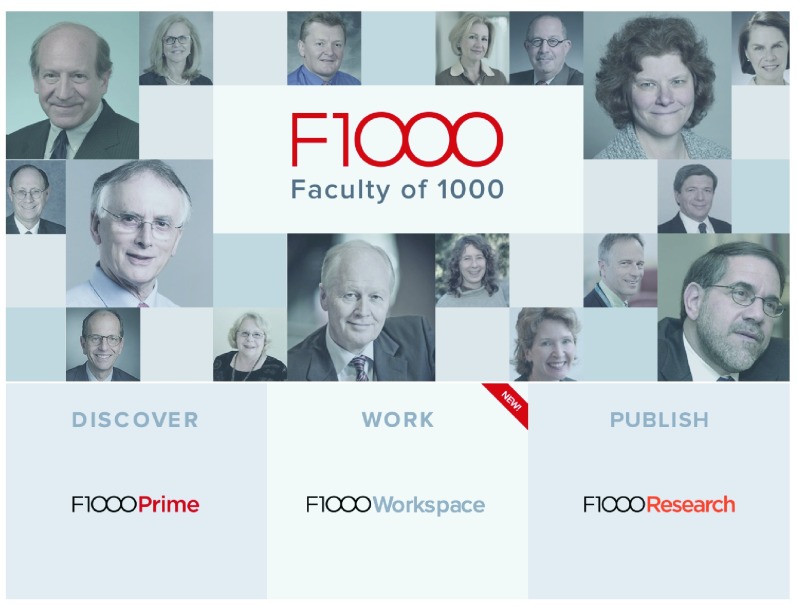
Faculty of 1000, which now comprises 3 core services: F1000Prime, F1000Workspace and *F1000Research*, and is overseen by the F1000 Faculty comprising over 11,000 members.

I founded Faculty of 1000, now renamed F1000 ( Figure 3) back in 2000. We are an organisation comprising many former scientists and a large number of software programmers. We have a history of innovation in science publishing, and we are deeply concerned about the harm science publishing has caused, and is causing science. We believe that our role in science publishing is to provide a service to researchers, who are the vast majority of the writers and readers of research articles. We believe that scientists have all the knowledge necessary to manage the decisions in this process, and that our role is to listen and to develop the tools and services to make that process work better.

To make sure we respond to genuine needs, and to avoid the many sins mentioned above, we have brought together a large group of leading researchers in biology and medicine, the F1000 Faculty, who act as our guides and critics, and who contribute directly to the services we provide in our post-publication peer review process. The Faculty has over 11,000 members comprising over 6000 senior researchers who are Faculty Members and about 5000 younger researchers acting as Associate Faculty Members.

### 1   F1000Prime – *qualitative assessment*

**Figure 4.**
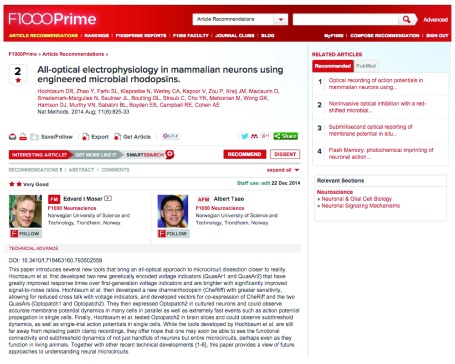
An example of an F1000Prime recommendation showing the Faculty Members who made the article recommendation, the rating they gave the article, and the associated comment as to why they felt that article was so interesting.

F1000Prime was developed to provide qualitative assessment of individual research articles by named experts. One of its goals is to act as one of the potential alternatives to the Impact Factor. The F1000 Faculty are asked to identify articles that they come across in their daily work that they find interesting. They rate them as one of three levels of quality (all positive; the goal is to find the best articles) and write a short recommendation explaining why the chosen articles are interesting to them ( Figure 4). To-date, we have over 160,000 such recommended articles, published in over 4,000 journals. In this way, the Faculty recommends about 1–2% of the biomedical literature in PubMed. Many new recommendations arrive every day.

F1000Prime serves to provide an effective way for a researcher to receive a frequent update of the papers that members of the Faculty found commendable in their field of research, or in a new field they need to explore. The assessment of the articles is not a reflection of the Impact Factor of the journals they were published in, but an individual assessment of the article by a named expert. In a sense, this is a type of post-publication peer review of the world’s scientific literature – the Faculty choose and comment on articles after they have been published.

### 2   F1000Research – *publishing platform*

**Figure 5.**
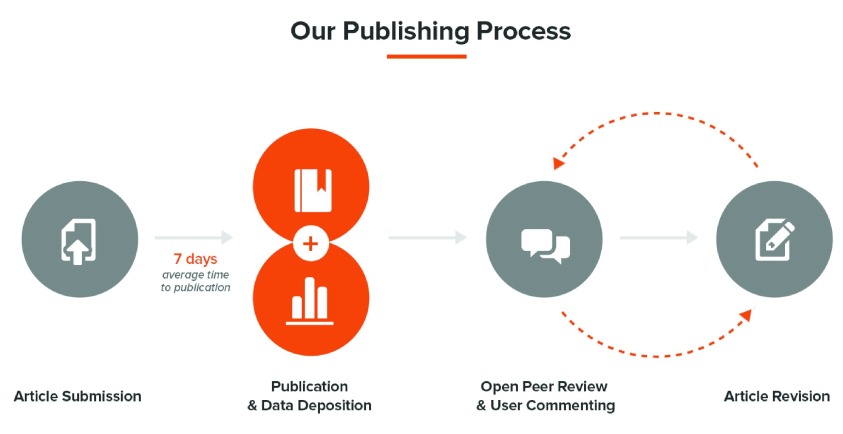
Summary of the *F1000Research* open science publishing process.

About two years ago, we started publishing research articles in a completely new way. *F1000Research* is an author-led publishing platform for biological and medical research that makes no editorial decisions, performs no secret refereeing, and removes the delay in publishing ( Figure 5). We are, of course, an open access publisher, and we encourage the publication of many article types, including short articles, negative findings, software articles, case histories, and many other valuable forms of research reporting currently shunned by many journals.

To avoid delay, we publish immediately. Following submission, we conduct a quick internal hygiene check: does the article come from a scientist in a scientific institution, does it look like a science article in its format, and does it conform to the ethical standards demanded of a biological or medical article? If these basic checks are passed, the article is then published and becomes citable, although clearly labelled as ‘awaiting peer review’.

**Figure 6.**
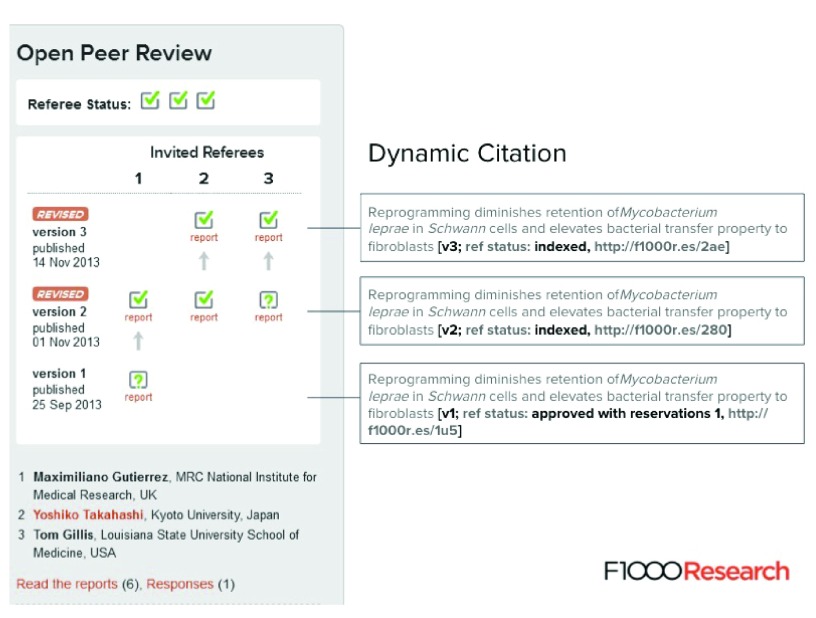
The Open Peer Review box that appears on all *F1000Research* articles that provides a summary of the peer review and version history of the article. It shows the different article versions, the names of the referees, the peer review statuses they have given the different article versions (green tick = Approved status; green question mark = Approved with Reservations status), and clickable links to the associated referee reports. Each article version is independently citable and includes details of the version number and referee status within the article title.

Publication then triggers peer-review; authors choose referees from a list derived from our Faculty at the time of submission. If they cannot find an appropriate expert in the subject of the article, they can make a suggestion and we verify the appropriateness of the referee. To ensure the peer review status of any article is always clear, we have developed a new dynamic citation format, now being introduced by PubMed and other bibliographic listing agencies, that includes both the version numbering and the refereeing status ( Figure 6).

Peer review is open, transparent and author-led: referees receive the request through us from the author, and are asked to make one of three determinations: approved, approved with reservations, or not approved. These are accompanied by a detailed peer review, which appears online alongside the article, together with the referee names and affiliations. The authors can publicly respond to the referee reports, and this commonly results in the submission of a revised article together with a short summary of the changes. The new version is published and independently citable. The referees respond to the new version, and this process continues until the authors do not find it necessary to provide a new version.

There is no ‘final article’ and nothing is ever removed, so readers can see the whole history and the conversation between the referees and authors. We make no editorial decisions, although our staff are always available with help and advice to both authors and referees. Once an article has passed peer review, it is listed in PubMed and other major bibliographic databases. We have found it surprisingly easy to find referees in this new process and they tend to write better comments, probably because their work is seen by readers. All referee reports are also made independently citeable, and are in fact cited. In this way, we believe that referees make a significant contribution to scientific discourse.

Finally, all of the data on which the article is based must be provided. We go to great lengths to ensure that we get the relevant data underlying the article, even if it is very large, and in a format that can be practically used, working with many groups to provide useful tools to view and manipulate different data types. We know that authors can sometimes be reluctant for different reasons to provide their data, but clearly neither referees nor readers can judge the veracity of findings without access to the data. There is an increasing suspicion that a large proportion of published research cannot be reproduced and verified, and we all need to work together to change that.

### 3   F1000Workspace – *tools for authors*

**Figure 7.**
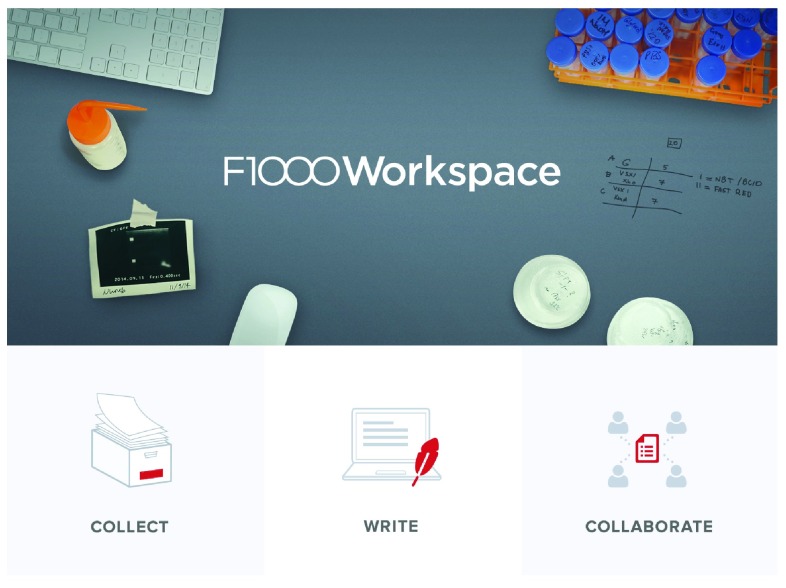
The new F1000Workspace that provides a set of tools to enable researchers to collect references, write research articles and grant applications, and collaborate with co-authors and colleagues.

Finally, we have just launched F1000Workspace, a set of tools designed specifically for researchers in biology and medicine, to help them to collect and manage references, annotate and collaborate with co-authors and colleagues, write research articles and grant applications and prepare them to meet specific journal and institutional requirements, and to submit them for publication ( Figure 7). We offer fast and effective assistance in identifying and managing references during the writing process, using algorithmic techniques to offer both the relevant references recommended by the Faculty, and incorporate other references from PubMed and elsewhere.

We are working on a special one-click process that enables authors to publish in *F1000Research*, where the article will become visible within a few days, after which the open and transparent refereeing starts. We believe the F1000Workspace will make a significant contribution to the process of preparing and writing research articles and other scientific documents.

## Becoming a good citizen

Our goal with the new F1000 integrated set of services is to work towards removing the sins that currently beset the communication and discussion of new scientific discoveries. I hope that striving for this goal will be proof of my full conversion from sinful publisher to good citizen of the research community.

